# Association of genetically determined chronotype with circulating testosterone: a Mendelian randomization study

**DOI:** 10.3389/fendo.2024.1264410

**Published:** 2024-04-26

**Authors:** Tomohiro Ichikawa, Takuro Kobayashi, Tsuyoshi Hachiya, Yoshihiro Ikehata, Shuji Isotani, Hisamitsu Ide, Shigeo Horie

**Affiliations:** ^1^ Department of Urology, Juntendo University, Graduate School of Medicine, Tokyo, Japan; ^2^ Department of Advanced Informatics for Genetic Diseases, Juntendo University, Graduate School of Medicine, Tokyo, Japan

**Keywords:** testosterone, sex steroid hormone, chronotype, sleep duration, Mendelian randomization (MR)

## Abstract

Low testosterone levels in men have been linked to decreased physical and mental function, as well as a reduced quality of life. Previous prospective observational studies have suggested an association between testosterone and sleep traits, but the causality of this relationship remains unclear. We aimed to explore the potential causal link between genetically determined sleep traits and testosterone levels in men using Mendelian randomization (MR) analysis from the UK Biobank dataset. Our exposures were genetic variants associated with sleep traits (chronotype and sleep duration), whereas our outcomes were traits of sex steroid hormones (total testosterone, TT; bioavailable testosterone, BAT; and sex hormone-binding globulin, SHBG). We employed inverse variance weighted (IVW) and weighted median (WM) methods to assess the causal associations. The IVW method offers a robust estimate of causality, whereas the WM method provides reliable results even when some genetic variants are invalid instruments. Our main analysis involving sex steroid hormones and chronotype identified 155 chronotype-related variants. The primary findings from the analysis, which used chronotype as the exposure and sex steroid hormones as the outcomes, showed that a genetically predicted chronotype score was significantly associated with an increased levels of TT (association coefficient β, 0.08; 95% confidence interval [CI], 0.02–0.14; *P* = 0.008) and BAT (β, 0.08; 95% CI, 0.02–0.14; *P* = 0.007), whereas there was no significant association with SHBG (β, 0.01; 95% CI, −0.02–0.03; *P* = 0.64). Meanwhile, MR analysis of sex steroid hormones and sleep duration was performed, and 69 variants associated with sleep duration were extracted. There were no significant association between sleep duration and sex steroid hormones (TT, *P* = 0.91; BAT, *P* = 0.82; and SHBG, *P* = 0.95). Our data support a causal association between chronotype and circulating testosterone levels in men. These findings underscore a potential causal relationship between chronotype and testosterone levels in men, suggesting that lifestyle adjustments are crucial for men’s health. Recognizing factors that influence testosterone is essential. One limitation of this study is the use of one-sample MR, which can introduce potential bias due to non-independence of genetic associations for exposure and outcome. In conclusion, our findings indicate that a morning preference is correlated with circulating testosterone levels, emphasizing the potential impact of lifestyle habits on testosterone levels in men.

## 1 Introduction

Testosterone, a key sex steroid hormone, is essential for muscle maintenance, hematopoiesis, and male sexual behavior in adult physiology (1–3). In men, approximately 95% is produced in the testes and the remaining 5% in the adrenal glands. Although it is produced in the ovaries and adrenal glands in women, the amount of production is much lower than that in men. Approximately 98% of total testosterone (TT) in the blood is bound to sex hormone binding globulin (SHBG) and albumin (as Alb-T), with the remaining 1%–2% present as free testosterone (FT). Among these, FT and Alb-T have biological activity as androgens, and thus, FT and Alb-T are collectively called bioavailable testosterone (BAT). On the other hand, SHBG has no biological activity and increases gradually with aging. Therefore, even if TT remains unchanged, BAT is thought to decrease relatively over an individual’s life (4). Low testosterone levels are associated with decreased physical and mental function and quality of life in men. Maintaining testosterone levels may contribute to extending healthy life expectancy (5–7).

Testosterone exhibits diurnal fluctuations (8). Similarly, sleep traits are also deeply affected by diurnal fluctuations. Therefore, the relationship with testosterone and sleep traits has recently attracted attention. Sleep traits, such as timing and duration of sleep, are varied greatly from person to person and are involved in the maintaining sleep homeostasis, such as 24-h internal rhythms that are synchronized with external environmental stimuli. Sleep traits are influenced by genetic factors, and disturbances in sleep traits have adverse effects on health (9). Timing of sleep, also called chronotype, expresses preferences for when to wake up and when to go to bed. Several observational studies reported the association of sleep traits and circulating testosterone levels. In a prospective observational study in Germany targeting college student volunteers (N = 106), *Randler et al.* used the composite scale of morningness to assess chronotype and sampled saliva testosterone in the midmorning to avoid seasonal and circadian effects. Morning preference was significantly associated with lower testosterone levels (r = −0.22; *P* = 0.023) compared with evening preference (10). Another prospective study in the United States targeted elderly volunteers (N = 12) aged 64–74 years. Three morning blood samples were pooled for the measurement of total and free testosterone. In addition to overnight laboratory polysomnography was used to determine the amount of nighttime sleep of the participants in everyday life settings. *Penev* reported that sleep deprivation was associated with lower testosterone levels (r = 0.842; *P* = 0.001) (11). However, these observational studies could not elucidate whether sleep habits causally influence testosterone levels, or testosterone levels affect sleep traits.

This study aimed to investigate potential causal associations between sleep traits (chronotype and sleep duration) and testosterone levels in men using Mendelian randomization (MR), which estimates the causal relationship between exposure factors and outcomes using genetic variants as instrumental variables (IVs) (12).

## 2 Materials and methods

### 2.1 Data source

**Figure 1:**
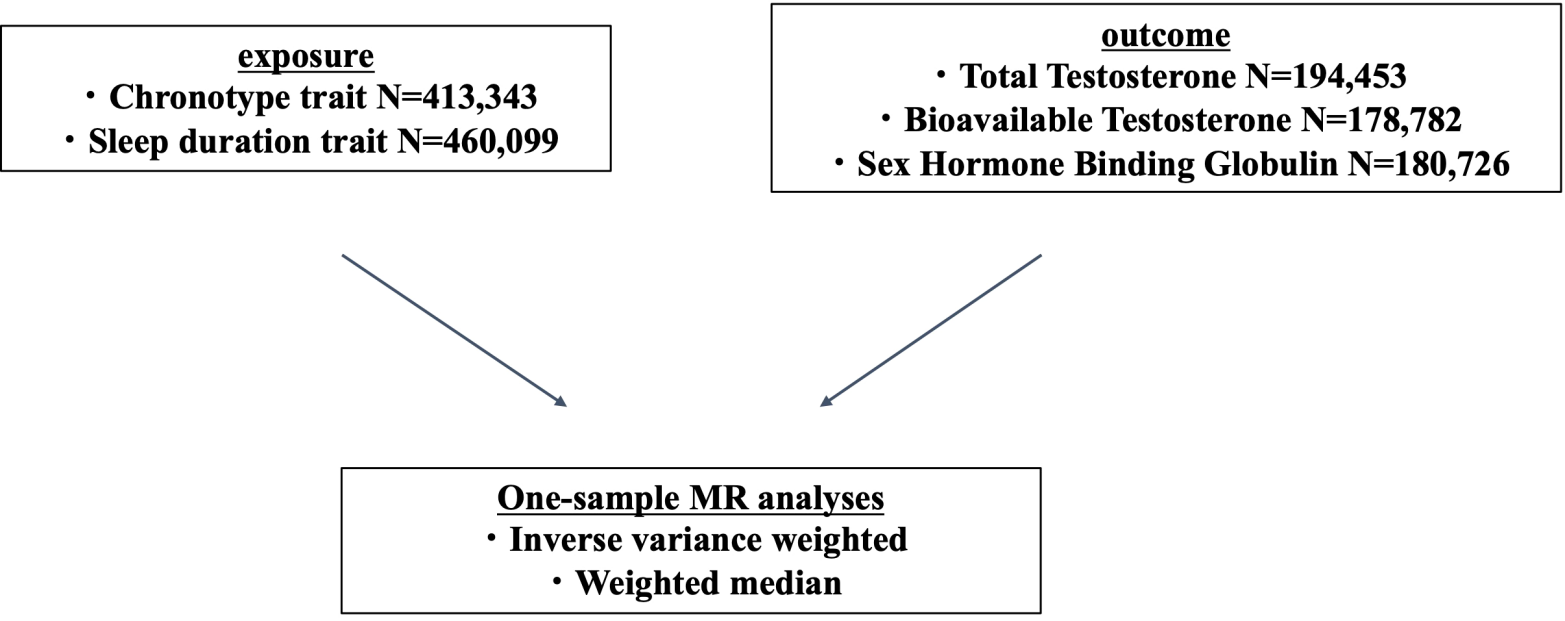
One-sample Mendelian randomization (MR) study design.

Using the genome-wide association studies (GWAS) database in the UK Biobank, we collected information on genetic variants associated with sleep traits and not associated with sex steroid hormones trait (13, 14). The UK Biobank is a large prospective cohort study with deep genetic and phenotypic data collected on approximately 500,000 individuals from across the United Kingdom, aged between 40 and 69 years at recruitment. Health-related information from each participant, including genetic and lifestyle information, blood and urine biomarkers, and physical characteristics, were collected. This database can be used as an open resource (15, 16). We performed MR analyses with sleep traits as exposure and sex steroid hormone levels as outcome (**Figure 1**) (17).

As sleep traits, information on chronotype (ID:ikb-b-4956) and sleep duration (ID:ukb-b-4424) was extracted. We analyzed 9,851,867 variants obtained from 413,343 individuals with chronotype information and extracted chronotype-associated variants based on the genome-wide significance level (P < 5×10^−8^). Chronotype was assessed using a questionnaire, “Do you consider yourself to be?” Possible answers were: “Definitely a ‘morning’ person”, “More a ‘morning’ than ‘evening’ person”, “More an ‘evening’ than a ‘morning’ person”, “Definitely an ‘evening’ person”, “Do not know,” and “Prefer not to answer”. The precomputed GWAS publicly available from UK Biobank defined the chronotype score as 1 for “Definitely a ‘morning’ person”, 2 for “More a ‘morning’ than ‘evening’ person”, 3 for “More an ‘evening’ than a ‘morning’ person”, and 4 for “Definitely an ‘evening’ person”. The continuous value of the chronotype score was modeled as outcome of linear regression with adjustment for sex and 10 principal components (PCs) calculated from the genotypes of the subjects. We analyzed 9,851,867 variants obtained from 460,099 individuals with information on sleep duration, and extracted associated SNPs (P < 5 × 10^−8^). Questions about average sleep duration were answered as integer numbers. The precomputed GWAS defined the sleep duration score as 1 for <7 h of sleep duration, 2 for 7 h–8 h, and 3 for ≥8 h. The continuous value of the sleep duration score was modeled as outcome of linear regression with adjustments for sex and 10 PCs (13).

Similarly, for sex steroid hormones, we extracted information on TT (ID:ebi-a-GCST90012113), BAT (ID:ebi-a-GCST90012103), and SHBG (ID:ebi-a-GCST90012109) in men. We investigate which components of circulating testosterone are associated with sleep traits. We analyzed 16,131,612 variants from 194,453 men with TT information, 16,131,701 variants from 178,782 men with BAT information, and 16,136,768 variants from 180,726 men with SHBG information. We extracted genetic variants that were strongly associated with each. In UK Biobank, blood samples were collected at initial visit. TT measured in nmol/L by one-step competitive analysis on a Beckman Coulter UniCel Dxl 800. The data were conducted inverse normal transformation of rank adjusted by fasting time, age, center, and chip/release of genetic data. SHBG measured in nmol/L by two-step sandwich immunoassay analysis on a Beckman Coulter UniCel Dxl 800. The data were conducted natural log transformation adjusted by age, dilution, batch, minutes since blood draw, time of blood draw, and operation status. Alb-T measured in g/L by BCG analysis on a Beckman Coulter AU5800. BAT was calculated from testosterone, accounting for concentration of SHBG and Alb-T using Vermeulen equation (14).

### 2.2 Statistical analysis

We performed MR analyses to assess the effects of the exposures (chronotype and sleep duration) on the outcomes (TT, BAT, and SHBG). Mendelian randomization uses genetic variants to determine whether an observational association between a risk factor and an outcome is consistent with a causal effect. Mendelian randomization relies on the natural, random assortment of genetic variants during meiosis yielding a random distribution of genetic variants in a population. Individuals are naturally assigned at birth to inherit a genetic variant that affects a risk factor or not inherit such a variant. Individuals who carry the variant and those who do not are then followed up for the development of an outcome of interest. Because these genetic variants are typically unassociated with confounders, differences in the outcome between those who carry the variant and those who do not can be attributed to the difference in the risk factor (12).

The causal association between exposure and outcome was estimated using inverse variance weighted (IVW) and weighted median (WM) methods as primary and secondary analyses, respectively (18–22). The IVW estimate is asymptotically equal to the two-stage least squares estimate commonly used with individual-level data. If the genetic variants are uncorrelated, and all genetic variants satisfy the IV assumptions, then the IVW estimate is a consistent estimate of the causal effect (18, 19).

The WM estimator is the median of a distribution having estimate percentile. For all other percentile values, we extrapolate linearly between the neighboring ratio estimates. The contribution of the genetic variant to the empirical distribution is proportional to its weight. The simple median estimator can be thought of as a weighted median estimator with equal weights. Although the simple median provides a consistent estimate of causal effect if at least 50% of IVs are valid, the weighted median will provide a consistent estimate if at least 50% of the weight comes from valid IVs. We assume that no single IV contributes more than 50% of the weight; otherwise, the 50% validity assumption is equivalent to assuming that this IV is valid (in which case, an analysis should simply be based on this one IV) (18, 19).

The MR-Steiger test was used to confirm the effect direction of causality. The MR-Steiger approach is used to determine the direction of a possible causal effect between two phenotypes. For two phenotypes, denoted phenotypes A and B, the MR-Steiger approach is composed of two parts. (i) MR is performed for a set of variants that serve as IVs for phenotype A. (ii) The difference of two correlations, the correlation between the variants and phenotype A and the correlation between the variants and phenotype B, is calculated. These two parts are then used to determine the direction of a possible causal effect between the two phenotypes (21, 22). Furthermore, MR-PRESSO was used to assess horizontal pleiotropy in the results that demonstrate causality (23, 24).

MR results were defined as statistically significant at two-sided *P<*0.05. We analyzed the relationship between three types of testosterone levels and each sleep trait. Therefore, Bonferroni correction was performed to correct the significance level (*P* < 0.016) due to multiple comparisons. All statistical tests were performed using the TwoSampleMR package version 0.5.6 and the R language version 4.2.1 (R Foundation for Statistical Computing, Vienna, Austria) (17).

## 3 Results

**Figure 2:**
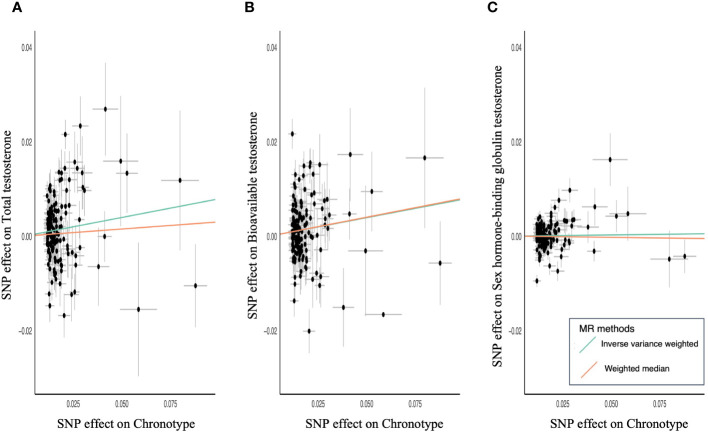
Scatter plots for MR analyses of chronotype and sex steroids hormone. **(A)** Total testosterone, **(B)** bioavailable testosterone, **(C)** sex hormone-binding globulin. Analyses were conducted using the IVW and WM methods using chronotype associated 155 variants.

MR of sex steroid hormones and chronotype was performed, and 161 variants associated with chronotype were extracted (**Supplementary Tables S1-S4**). One was not included in the GWAS of sex steroid hormones, and five palindromic variants were excluded. Thus, we extracted the association statistics for the remaining 155 chronotype-related variants, which were used as IVs for MR analysis. In the primary analysis of IVW, genetically predicted chronotype score was significantly associated with an increased level of TT (β = 0.08; 95% confidence interval [CI], 0.02–0.14; *P* = 0.008) (**Table 1**; **Figure 2A**). Similarly, genetically predicted chronotype score was significantly associated with an increased level of BAT (β = 0.08; 95% CI, 0.02–0.14; P = 0.007, **Table 1**; **Figure 2B**). No significant association was observed between chronotype score and SHBG (β = 0.01; 95% CI, −0.02–0.03; *P* = 0.64) (**Table 1**; **Figure 2C**). The results suggest that morning preference was shown to have lower TT and BAT.

**Table 1:** MR analyses of chronotype and sex steroids hormone.

Exposure	Outcomes	MR methods			
		Inverse variance weighted		Weighted median test	
		Beta (95% CI)	P-value	Beta (95% CI)	P-value
**Chronotype** **(155 variants)**	**Total testosterone**	0.08 (0.02–0.14)	0.008	0.03 (−0.03–0.09)	0.32
	**Bioavailable testosterone**	0.08 (0.02–0.14)	0.007	0.08 (0.02–0.14)	0.006
	**Sex hormone-binding globulin**	0.01 (−0.02–0.03)	0.64	−0.01 (−0.03–0.02)	0.65

The relationship between chronotype and TT showed a similar tendency in the secondary analysis of WM (β = 0.03; 95% CI, −0.03–0.09; *P* = 0.32) (**Table 1**). MR-PRESSO revealed horizontal pleiotropy (global test P < 0.001) and showed that nine out of 155 variants were outliers. Chronotype demonstrated a significant causal effect on TT in the outlier-corrected estimate (β = 0.06; 95% CI, 0.01–0.11; P = 0.013). The relationship between chronotype and BAT using WM analysis also showed a significant association (β = 0.08; 95% CI, 0.02–0.14; P = 0.006) (**Table 1**). MR-PRESSO revealed horizontal pleiotropy (global test P < 0.001) and determined that seven out of 155 variants were outliers. Chronotype exhibited a trend of causal effect on BAT even in the outlier-corrected estimate (β = 0.05; 95% CI, 0.004–0.10; P = 0.034). In both analyses, the MR-Steiger test confirmed that chronotype influenced TT and BAT in the positive direction of causality.

To confirm the absence of reverse causality, a similar analysis was performed with sex steroid hormones as the exposure and chronotype as the outcome. As a result, no significant association was observed between the two (TT: β = −0.01; 95% CI, −0.03–0.01; P = 0.18, BAT: β = −0.006; 95% CI, −0.03–0.02; P = 0.67, SHBG: β = −0.01; 95% CI, −0.04–0.02; P = 0.55). Therefore, it was shown that testosterone levels do not affect chronotype, confirming that there is no reverse causal relationship.

**Figure 3:**
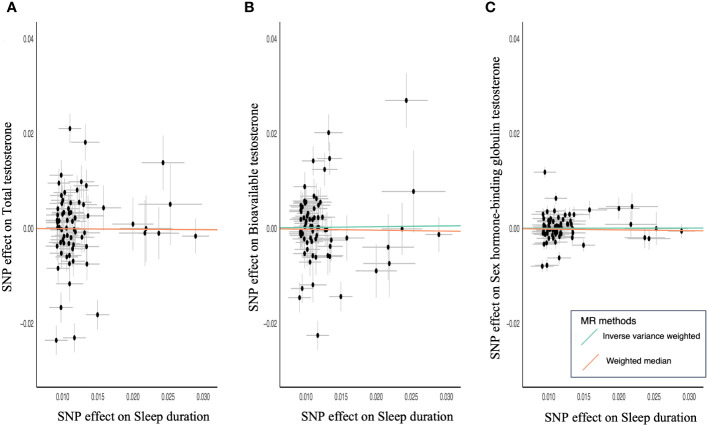
Scatter plots for MR analyses of sleep duration and sex steroids hormone. **(A)** Total testosterone, **(B)** bioavailable testosterone, and **(C)** sex hormone-binding globulin. Analyses were conducted using the IVW and WM methods using sleep duration associated 69 variants.

MR of sex steroid hormones and sleep duration was performed, and 71 variants strongly associated with sleep duration were extracted (**Supplementary Tables S5-S8**). Two palindromic variants were excluded. Thus, we extracted the association statistics for the remaining 69 sleep duration-related variants, which were used as IVs for MR analysis. In the primary analysis of IVW, an increase in genetically predicted sleep duration score was not significantly associated with TT (β = −0.01; 95% CI, −0.17–0.15; *P* = 0.91) (**Table 2**; **Figure 3A**). Similarly, no significant association was observed between sleep duration and BAT (β = 0.02; 95% CI, −0.13–0.06; *P* = 0.82) (**Table 2**; **Figure 3B**). The results were similar for SHBG (β = 0.002; 95% CI, −0.06–0.06; *P* = 0.95) (**Table 2**; **Figure 3C**).

**Table 2:** MR analyses of sleep duration and sex steroids hormone.

Exposure	Outcomes	MR methods			
		Weighted inverse variance		Weighted median test	
		Beta (95% CI)	P-value	Beta (95% CI)	P-value
**Sleep duration** **(69 variants)**	**Total testosterone**	−0.01 (−0.17–0.15)	0.91	−0.01 (−0.13–0.11)	0.88
	**Bioavailable testosterone**	0.02 (−0.13–0.16)	0.82	−0.02 (−0.15–0.11)	0.76
	**Sex hormone-binding globulin**	0.002 (−0.06–0.06)	0.95	−0.02 (−0.06–0.03)	0.49

To confirm an inverse causal association, a similar analysis was performed using sex steroid hormones as the exposure and sleep duration as the outcome. TT, BAT, and SHBG were not significantly associated with sleep duration (TT: β = −0.01; 95% CI, −0.03–0.002; P = 0.09, BAT: β = −0.01; 95% CI, −0.03–0.01; P = 0.31; SHBG: β = −0.03; 95% CI, −0.05 to −0.002; P = 0.03). It became clear that there was no causal relationship between testosterone levels and sleep duration.

A causal relationship between testosterone levels and chronotype was confirmed, but not with sleep duration. Interestingly, different results were shown depending on sleep traits.

## 4 Discussion

Previous several observational studies have been reported between sleep traits (chronotype, sleep duration) and circulating testosterone levels (8, 10, 11), but all were small population and the causal association was unclear. We analyzed the causal association between sleep traits and testosterone using MR. Our data showed that morning preference was causally associated with lower testosterone levels (TT and BAT), whereas there was no significant association between sleep duration and testosterone.

Testosterone is highest after waking up and decreases toward night (25). Since more testosterone is produced during sleep, morning preference may be starting activity before they have enough testosterone produced. A latent state of low testosterone may have a negative impact on the maintenance of muscle mass and strength, hematopoiesis, and male sexual function.

Testosterone replacement therapy has previously been reported as a treatment for low testosterone in men. A 3-year study of testosterone patch supplementation in men aged 65 years and over in the United States found effects on improving body composition, such as increasing muscle mass and decreasing fat mass (26). On the other hand, it has been reported that in the group, a decrease in testosterone due to antiandrogen therapy including GnRH agonist administration in patients with prostate cancer and changes in body composition such as decreased muscle mass and increased fat mass were observed (27). In a meta-analysis examining the efficacy of testosterone replacement therapy for sexual function, the therapy showed modest improvements in the number of nocturnal erections, erectile function scores, and overall sexual satisfaction in men with low mean testosterone levels (28).

The chronotype has been associated with individual differences in traits related to circadian rhythms, such as body temperature, cortisol, and melatonin secretion. A previous MR study using dataset from the UK Biobank and PRACTICAL Consortium reported that morning preference was associated with a decreased risk of developing prostate cancer (29). A meta-analysis of 20 prospective studies reported that low free testosterone levels were associated with a lower risk of developing prostate cancer (30). Yet, another MR study reported that morning preference was associated with a decreased risk of breast cancer (31). This study and the results of previous studies suggest, although not directly, that individuals with morning preference may tend to have lower testosterone levels; similar findings on sex steroid hormones and sleep habits using two-sample MR have recently been reported (32), which in turn may reduce the risk of developing prostate cancer. Our application of one-sample MR to the association between testosterone and sleep allowed us to validate previous findings and check robustness, ensuring that the observed associations were consistent across different methodologies. This strengthened the reliability of the results, provided a complementary perspective, and reduced potential biases inherent in each methodology.

Focusing on aspects other than testosterone, evening preference is more likely to be associated with mental disorders (OR, 1.94; P < 0.001), diabetes (OR, 1.30; P < 0.001), neuropathy (OR, 1.25; P < 0.001), gastrointestinal/abdominal disorders (OR, 1.23; P < 0.001), respiratory disorders (OR, 1.22; P < 0.001), and increased risk of all-cause mortality (HR, 1.10; P = 0.012) (33). Another previous report that those with evening preference had lower adherence to the Baltic Sea diet score and were more often smokers (men), were physically inactive, and had lower perceived health than those of other chronotypes (P < 0.05) (34). Furthermore, those with evening preference experienced insomnia symptoms, had nightmares, and had used recently hypnotics significantly more often than other chronotypes among both men and women (35). These previous reports suggest that regarding lifestyle guidance for patients with low testosterone levels, caution should be practiced regarding recommending evening preference, taking into consideration the effects that lifestyle can have on the mind and body.

*Montaruli et al.* report that the circadian rhythm plays a fundamental role in regulating biological functions, including sleep–wake preference, body temperature, hormonal secretion, food intake, and cognitive and physical performance. Circadian rhythm alterations could create predispositions to the development of several diseases (such as cancer and neurodegenerative, cardiovascular, and metabolic diseases) and to physiological aging. Interindividual differences in chronotypes need to be considered to reduce the negative effects of circadian disruptions on health (36). Therefore, we need to further understand the true role of chronotype.

In understanding diseases, it is extremely important to clarify their causes and causal relationships. By identifying causal relationships, it becomes easier to consider ways to prevent and deal with diseases. Essentially, in order to make causal inferences, it is necessary to investigate causal relationships by eliminating confounding effects through randomized clinical trials. However, conducting randomized clinical trials requires many resources. MR analysis has the great advantage of being able to make causal inferences that minimize the effects of confounding by taking advantage of the fact that genetic polymorphisms are randomly inherited by offspring (12).

However, this study has several research limitations. First, we used an analysis method called one-sample MR. One-sample MR is a method of estimating causality using GWAS results obtained from one population for exposure and outcome, whereas two-sample MR assumes that association of genetic variants with exposure and outcome variables was estimated using independent populations. In contrast to two-sample MR, one-sample MR derives both estimates from the same cohort, leading to potential violation of the independence assumption when confounding is present. As a result, one-sample MR may introduce potential bias. A recent study assessing the bias and accuracy of causal inference for one- and two-sample MR has suggested that one-sample MR yields unbiased estimates when using large-scale cohorts such as the UK Biobank. In this study, we applied the one-sample MR to the UK Biobank datasets (20), enabling a large-scale analysis. Second, our analysis failed to consider potential genetic pleiotropy. Therefore, supplementary analyses that accommodate the potential genetic pleiotropy are warranted (37). Additionally, to solidify the causal relationship between testosterone and sleep traits, future investigations employing different MR analytical techniques beyond the ones used in this study are essential. Third, we used the European-ancestry dataset for MR analyses (13, 14). It is unclear whether the present results can be applied to other ancestry. Further studies are needed to determine whether our results can be extrapolated across ancestries. Finally, the chronotype score and sleep duration were adjusted for sex and 10 PCs, but not for age. Further studies are needed to clarify the age-dependent causal relationship between the chronotype score and testosterone levels.

In conclusion, we showed that morning preference was causally associated with a decreased level of circulating testosterone. This result reinforces those shown in previous observational studies. However, the exact reason why chronotype affects testosterone is not clear, and this remains a topic for future research.

## Data availability statement

Publicly available datasets were analyzed in this study. This data can be found here: https://gwas.mrcieu.ac.uk; Dataset:ebi-a-GCST90012113,ebi-a-GCST90012103,ebi-a-GCST90012109,ukb-b-4956,ukb-b-4424.

## Author contributions

TI: Conceptualization, Data curation, Formal Analysis, Investigation, Writing – original draft, Writing – review & editing. TK: Data curation, Formal Analysis, Investigation, Resources, Validation, Visualization, Writing – original draft, Writing – review & editing. TH: Data curation, Formal Analysis, Investigation, Resources, Validation, Visualization, Writing – review & editing. YI: Investigation, Project administration, Validation, Writing – review & editing. SI: Investigation, Validation, Writing – review & editing. HI: Supervision, Validation, Writing - review & editing. SH: Conceptualization, Project administration, Supervision, Writing – review & editing.
